# Cross-Talk Between Antigen Presenting Cells and T Cells Impacts Intestinal Homeostasis, Bacterial Infections, and Tumorigenesis

**DOI:** 10.3389/fimmu.2019.00360

**Published:** 2019-03-06

**Authors:** Stephen J. Gaudino, Pawan Kumar

**Affiliations:** Department of Molecular Genetics and Microbiology, Stony Brook University, Stony Brook, NY, United States

**Keywords:** APC, SFB, CRC, SCFA, TLR, PAMP

## Abstract

Innate immunity is maintained in part by antigen presenting cells (APCs) including dendritic cells, macrophages, and B cells. APCs interact with T cells to link innate and adaptive immune responses. By displaying bacterial and tumorigenic antigens on their surface via major histocompatibility complexes, APCs can directly influence the differentiation of T cells. Likewise, T cell activation, differentiation, and effector functions are modulated by APCs utilizing multiple mechanisms. The objective of this review is to describe how APCs interact with and influence the activation of T cells to maintain innate immunity during exposure to microbial infection and malignant cells. How bacteria and cancer cells take advantage of some of these interactions for their own benefit will also be discussed. While this review will cover a broad range of topics, a general focus will be held around pathogens, cancers, and interactions that typically occur within the gastrointestinal tract.

## Introduction to Innate Immunity, APC Activation, and T Cell Function

The immune system is divided into innate and adaptive responses. Adaptive immunity is regulated by B cells and T cells. Maturation of T cells occurs in the thymus and maturation of B cells occurs in the bone marrow. During antigen-dependent activation, B cells can develop into memory cells, which are activated upon subsequent exposure to the contacted antigen, or plasma cells, which secrete antibodies specialized to target that antigen (1). Similarly, T cells can develop into memory cells or effector cells. The two major types of effector T cells produced by the adaptive immune system are helper T cells (T_h_) and cytotoxic T cells (T_C_). T_h_ cells are distinguished by their expression of CD4, subset-specific expression of transcription factors (T-bet, GATA3, and RORγt), and the release of cytokines that influence the activation and differentiation of other immune cells. Three major subsets of T_h_ cells exist (T_h_1, T_h_2, and T_h_17), each of which is specialized for protecting against certain infections. T_h_1 cells primarily secrete interferon-γ (IFN-γ) which is associated with protection against intracellular microbes (predominantly viruses) and the onset of anti- or pro-tumorigenic effects, T_h_2 cells fight parasitic infections by secreting specific interleukin (IL) proteins including IL-4, IL-5, and IL-13, and T_h_17 cells fight microbial pathogens by secreting cytokines such as IL-17A, IL-17F, and IL-22 (2–6). T_C_ cells are distinguished by their expression of CD8 and their ability to directly contact and kill transformed and infected cells (1, 7). T cells and B cells act together to establish an immunological memory against individual pathogens or cancer cells. Adaptive immunity can take a few days to fully develop, but once activated allows for the mounting of a rapid immune response upon subsequent exposures to the specific pathogen or cancer cell. Innate immunity, unlike adaptive immunity, is traditionally not based on immunological memory. However, certain innate cells, particularly natural killer (NK) cells, aid in the development of immunological memory against viruses. For instance, Ly49H^+^ NK cells exposed to mouse cytomegalovirus (MCMV) expand to mount a primary immune response (8). This process is promoted by IL-18 signaling (9). Naïve mice that received an adoptive transfer of memory NK cells were able to mount a strong secondary response upon infection with MCMV (8). Signaling by IL-18, however, is not required for recall responses by memory NK cells (9).

Classical antigen presenting cells (APCs) are dendritic cells (DCs) and B cells (10). To mount an immune response, APCs must first recognize and bind their target. To do so, APCs express antigen-specific surface receptors including pattern recognition receptors (PRRs). PRRs detect pathogen-associated molecular patterns (PAMPs), which are produced by microbes, and damage-associated molecular patterns (DAMPs), which are produced by damaged or mutated host cells (11). Depending on the receptor, expression of PRRs can be constitutive or inducible (12, 13). One major family of PRR is the Toll-like receptors (TLRs). TLRs are typically expressed on the cell surface or within endosomes and are type I transmembrane proteins whose extracellular domains express leucine-rich repeats that are used to recognize and bind to specific PAMPs (14–16). Once the extracellular domain binds its target, the TLR activates a cytosolic signaling cascade which is initiated by an adaptor protein that interacts with the intracellular domain of the TLR. Depending on the TLR, the two adaptor sets that can be activated are TIRAP-MyD88 and TRAM-TRIF (14, 16–18). Another group of PRR is the nucleotide binding oligomerization domain (NOD)-like receptors (NLRs). NLRs are present in the cytoplasm and, like TLRs, initiate signaling cascades upon binding to microbial PAMPs (14, 16). After binding to their appropriate PAMP or DAMP, APCs internalize their target by initiating phagocytosis, pinocytosis, or clathrin-mediated endocytosis. The pathway by which molecules are endocytosed determines how they will be degraded and then displayed by major histocompatibility complex (MHC) for T cell recognition (19, 20).

Two types of MHCs display antigens: class I MHCs and class II MHCs. While MHC I receptors are produced by all nucleated cells and display endogenous antigens to activate CD8^+^ T_C_, only APCs produce MHC II receptors to display exogenous antigens and activate CD4^+^ T_H_ cells. Some APCs including DCs can also present exogenous antigens to the MHC I receptor to activate CD8^+^ T cells during a process called cross-presentation (20–23). Presentation of antigens by either MHC I or MHC II receptors also depends on antigen composition (particulate vs. soluble), method of endocytosis, and degradation by lysosomal proteases (20). T_C_ and T_H_ cells utilize membrane-bound T cell receptors (TCRs) to bind MHC receptors (1). TCRs consist of two polypeptide chains (alpha and beta) that are linked together by disulfide bonds. T cells also produce co-receptors on their surface which further aid stabilizing interactions with MHCs of APCs. These include CD4 and CD8 (11, 20, 24). Costimulatory interactions between APCs and T cells can also occur, respectively, between B7 and CD28, ICAM-1 and LFA-1, and CD40 and CD40L (25, 26).

## Immunity to Bacteria

In this section, innate immunity will be discussed with respect to commensal and pathogenic bacteria. First, immunity will be reviewed within the context of commensals and their influence on altering the activity of T_h_17 and T regulatory (T_reg_) cells. Then, APC interactions with T cells, mainly regarding the expression of various TLRs, to fight pathogenic infections will be discussed.

## Immunity and Bacterial Commensals

The gut microbiota is composed of all the archaea, fungi, protozoans, viruses, and bacteria that inhabit the gastrointestinal tract. Specifically, the colon of a healthy 29 to 30-year-old male of average weight and height has been estimated to contain ~ 3.8 × 10^13^ bacteria (27). Commensal bacteria have been shown to modulate immune cell responses in the intestine. Reciprocally, immune cells interact with intestinal epithelial subsets to regulate colonization by commensal bacteria. Altered microbiota composition and aberrant immune responses to commensal bacteria, however, have been thought to play a role in the development of metabolic disorders (obesity and type II diabetes), autoimmune disorders (multiple sclerosis (MS) and type I diabetes), and inflammatory bowel disease (IBD) (28–31).

APCs, specifically DCs and macrophages, utilize PRRs to maintain intestinal homeostasis (discussed in “Immunity and Bacterial Pathogens” section). Macrophages plays an important role in maintaining tolerance against the commensal microbiota and food antigens. Although macrophages display robust bactericidal activity, they are not a key source of major inflammatory cytokines such as TNF-α, IL-1β, IL-6, and IL-23 (32, 33). However, macrophages constitutively produce and respond to IL-10. Macrophage-derived IL-10 is critical for Foxp3^+^ T_reg_ cell development, maintenance, and expansion (34–36). DCs also maintain tolerogenic responses by interacting with adaptive immune cells. DCs presents luminal antigens in secondary lymphoid organs of the intestine. These include the Peyer's patches (PPs) and mesenteric lymph nodes (MLNs). DCs regulate the homing of lymphocytes to the intestine by inducing expression of gut homing receptors (CCR9 and α4β7) (37, 38). DCs also are the major source of IL-23 which, in combination with other cytokines, influences the differentiation of T_h_17 cells and promotes the generation of IL-22, a tissue protective cytokine. Defects in NOD2 and autophagy-related genes (*ATG16L* and *IRGM*) in DCs of IBD patients reveal compromised antigen presentation, cytokine release, and increased inflammation (39–41).

IBD is marked by chronic gastrointestinal inflammation due to the onset of disorders such as ulcerative colitis (UC) and Crohn's disease (CD). Increased expression of T_h_1-derived IFN-γ and T_h_17 associated IL-17A and IL-22 are evident in the inflamed tissue of CD patient (42, 43). Genome-wide association studies show that genes (*IL23R* and *STAT3*) encoding T_h_17 differentiation pathways are associated with increased IBD risk. This suggests a therapeutic significance of targeting IL-23 or IL-17A in IBD (44, 45). Indeed, neutralizing IL-23 has been shown to be effective in reducing intestinal inflammation. Ustekinumab, an antibody directed against the IL-12p40 subunit, is used to block both IL-12 and IL-23 and is approved by the FDA for treatment of moderate to severe CD, whereas antibody targeting IL-23p19 subunit (IL-23) currently is in clinical trial for both CD and UC (46, 47). Interestingly, a clinical trial in which an anti-IL-17A monoclonal antibody was used to treat CD was prematurely ended since treatment with these antibodies was associated with detrimental effects (48). Subsequently we and others have shown that IL-17A is critical for preserving the epithelial barrier and regulating gut microbiota colonization (30, 49, 50). Additionally, it has been shown that IL-23 is not required for IL-17A generation from γδ T cells during chemical-induced colitis (49). This may explain why anti-IL-23 and IL-17A neutralization have counterintuitive outcomes. It remains unclear what regulates IL-17A generation in γδ T cells. It is possible that DC-derived IL-6 or other factors regulate IL-17A responses in the gut since CD103^+^ CD11b^+^ DCs have been shown to regulate T_h_17 differentiation in an IL-16 dependent manner (51).

The gut microbiota and microbial metabolites have been shown to regulate IL-17A responses in the gut. The intestinal microbiota, particularly segmented filamentous bacteria (SFB), has been shown to induce T_h_17 responses (52–54). Induction of T_h_17 cell responses by SFB is well-characterized. Monocyte-derived macrophages regulate SFB induction of antigen-specific T_h_17 cells (55, 56). Furthermore, colonization by SFB is associated with increased expression of IL-21 and isoforms of serum amyloid A (SAA). SAA indirectly promotes differentiation of T_h_17 cells by acting on DCs (53, 57). Along with SFB, a mixture of twenty bacterial isolates including *Clostridium* and *Bifidobacterium* species from an ulcerative colitis patient has been shown to induce T_h_17 activity (57). Additionally, *Escherichia coli Schaedler* and *Morganella Morganii* have been shown to regulate T_h_1 and T_h_17 cell differentiation via monocyte-derived DCs (58). Moreover, CD172α^+^ lamina propria DCs promote microbial antigen-specific T_h_17 cell differentiation in responses to TLR5 activation (59). The microbiota, including SFB, induces T_h_17 responses; however, it is poorly understood how immune cells regulate functions of the gut microbiota such as colonization by SFB. We and others have shown that IL-17A and IL-22 regulate the gut microbiota, including SFB colonization (30, 60, 61). Furthermore, we show that intestinal regulation of the gut microbiota by IL-17A modulates systemic autoimmunity suggesting a yin-yang relationship between the gut microbiota and T_h_17 cell responses (30).

The differentiation of naïve T cells into pathogenic (α/β CD4^+^ T cells that express high levels of IL-23R, coproduce IL-17A and IFN-γ/GM-CSF and induce autoimmunity) or non-pathogenic (α/β CD4^+^ T cells that produce IL-17A and IL-17F but do not induce autoimmunity) T_h_17 cells is influenced by DC-derived cytokines. Naïve T cells exposed to TGF-β1 and IL-6 differentiate into non-pathogenic T_h_17 cells, but those exposed to TGF-β1, IL-6, and IL-23 or TGF-β3 and IL-6 develop into pathogenic T_h_17 cells (62). Signaling by IL-23 increases expression of T-bet and production of TGF-β3 by developing T_h_17 cells. Likewise, IL-23 signaling has been associated with increased expression of RORγt and production of GM-CSF, an essential cytokine for the progression of autoimmunity, by T_h_17 cells (63). Production of dietary-derived fatty acid metabolites has also been shown to alter the differentiation of T cells (64). For instance, stimulation by long chain fatty acids triggers naïve T cell differentiation into T_h_1 and T_h_17 cells via the upregulation of p38-MAPK. This, in turn, promotes the onset of autoimmunity (64).

While SFB have mainly been associated with T_h_17 cell differentiation, *Bacteroides fragilis* or Clostridia species have been shown to regulate the induction and activity of T_reg_ cells (65, 66). Polysaccharide A derived from *B. fragilis* activates DCs in a TLR2-dependent manner to induce T_reg_ cell differentiation and IL-10 generation (66, 67). A mixture of seventeen Clostridia species that induce T_reg_ cell differentiation and function were isolated from a human fecal sample (65). When germ-free mice were inoculated with the mixture, an increase in T_reg_ cell abundance and induction were observed. These changes may be due to an increased production of microbiota-dependent fatty acid metabolites, particularly SCFAs. This study shows that SCFAs stimulate secretion of TGF-β by epithelial cells to promote induction of T_reg_ cells (65). Kashiwagi et al show that TGF-β derived from DCs via TLR2-Smad3 pathways is important for the generation of T_reg_ cells in the lamina propria of mice that were inoculated with *Clostridium butyricum* (68). Subsequently, the importance of SCFAs particularly butyrate in regulating T_reg_ differentiation has been shown by many studies (69, 70). Butyrate and propionate have been shown to directly modulate T_reg_ generation by promoting histone H3 acetylation of the Foxp3 locus and protein (69, 70). Additionally, butyrate signaling in macrophages and DCs via GPR109a, a receptor for butyrate and niacin, has been shown to promote T_reg_ cell development (71). Mice deficient in GPR109a have fewer IL-10 producing CD4 T cells (71). Colonic T_reg_ cells express TCRs, including CT7, that most likely aid in the recognition of specific antigens derived from the commensal microbiota (72). These TCRs are unique to colonic T_reg_ cells since they are not expressed by T_reg_ cells outside the colon (72).

APCs also modulate commensal microbiota-dependent T_h_2 cell responses. Mice treated with propionate display enhanced production of macrophage and DC precursors in their bone marrow. However, these DCs are impaired in eliciting effector functions of T_h_2 cells in a house dust mite extract-dependent allergic inflammation model (73).

**Figure 1:**
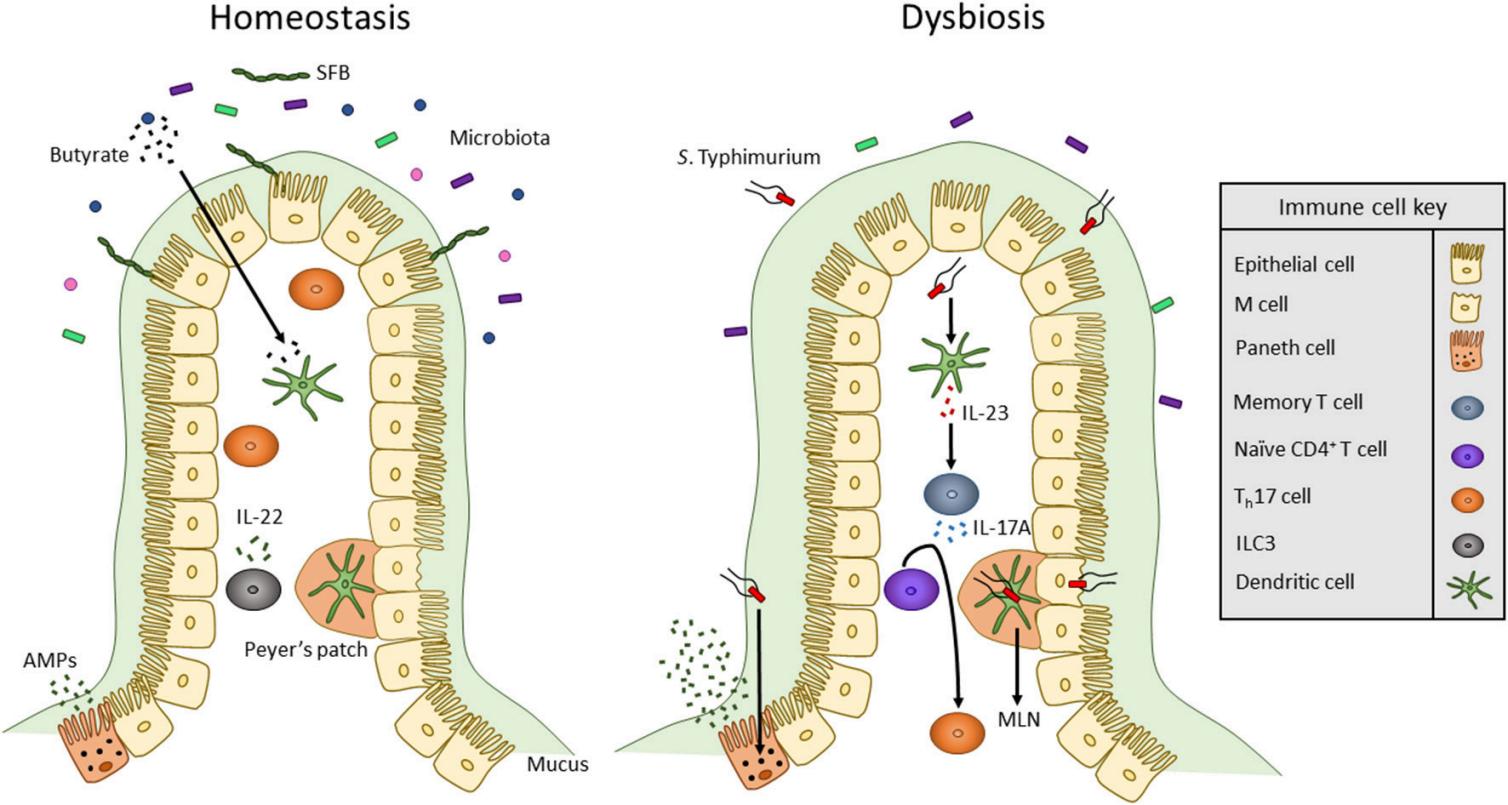
Overview of innate immune interactions under homeostasis and dysbiosis. In healthy individuals, the commensal microbiota secretes products, including SCFAs such as butyrate, that act on DCs to maintain a commensal-tolerant immune system. The presence of commensal microbes has also been associated with an increased presence of T_h_17 cells. Also, ILCs secrete cytokines to alter T cell function to maintain homeostasis. When dysbiosis is induced by bacterial pathogens such as *S*. Typhimurium, microbiota composition changes and mucus secretion increases. *S*. Typhimurium infection occurs via transcytosis by M cells into Peyer's patches where the bacterium can travel through lymphatic vessels to the MLN. To mount an immune response, *S*. Typhimurium antigens such as flagellin monomers are detected by TLR5 of DCs. DCs then produce IL-23 which promotes the release of IL-17 by memory T cells. IL-17 stimulates the differentiation of naïve CD4^+^ T cells into T_h_17 cells. Likewise, TLR9 and NOD2 receptors are activated within Paneth cells to upregulate the secretion of AMPs.

Along with T_h_17 and T_reg_ cells, innate lymphoid cells (ILCs) maintain immunity by interacting with APCs to influence commensal bacteria and T cell effector functions. ILCs are separated into three groups (ILC1, ILC2, and ILC3) based partially on the cytokines they secrete. Similar to T_h_17 cells, ILC3 cells secrete IL-17A and IL-22 (Figure 1) (74). IL-22 secreted from ILC3 can act on epithelial cells to induce expression of antimicrobial peptides. IL-23 derived from CD103^+^ CD11b^+^ DCs has been shown to regulate innate IL-22 responses following administration of bacterial flagellin (75). ILC3s also directly interact with T cells. MHC II receptors are expressed by CCR6^+^ RORγt^+^ ILCs which allows for direct binding and presentation of antigens to CD4^+^ T cells (76). Upon interacting with T cells, intestinal ILCs maintain homeostasis by limiting immune responses against commensal bacteria (76). Mononuclear phagocyte-derived TNF-like ligand 1 (TL1A) has been shown to regulates ILC3-dependent regulation of IL-22 production and mucosal host defense during acute colitis (77). TL1A also regulates expression of costimulatory molecule OX40L in MHC II^+^ ILC3s. This is required for antigen-specific T cell responses in a chronic colitis model (77).

## Immunity and Bacterial Pathogens

While commensal bacteria directly or indirectly (via APCs) influence the differentiation of T cells, APCs utilize TLRs to recognize specific microbial markers alter the function of T cells to fight bacterial pathogens. Cell surface TLRs aid in the phagocytosis of microbial pathogens by DCs and macrophages. Binding of surface TLRs to specific microbial PAMPs or whole bacteria such as *E. coli* or *Staphylococcus aureus* induces receptor phagocytosis (78). Activation of different APC TLRs results in the production of cytokines that promote the differentiation of naïve T cells into T_h_1 cells. This is evidenced by the activation of two TLRs, TLR2 and TLR4, that are expressed in immune cells and intestinal epithelial cells (79). TLR2 binds to Gram-positive and Gram-negative bacterial components including lipoteichoic acid, porins, and peptidoglycan, but TLR4 binds only to lipopolysaccharide, a component specific to the Gram-negative outer membrane (79–81). When activated, TLR4 promotes the production of IL-12 p70, the active form of IL-12 which is composed of the p35 and p40 subunits. IL-12 p70 aids in the polarization of naïve T cells into T_h_1 cells (81). On the other hand, activation of TLR2 results in increased production of IL-12 p40 homodimer which acts as a receptor antagonist of IL-12 (81).

A third TLR expressed by immune cells and the intestinal epithelium is TLR5, a receptor that specifically binds to flagellin monomers of Gram-positive and Gram-negative bacteria (79). Flagella are long whip-like structures utilized by bacteria for motility, adhesion, and secretion of virulence factors. Numerous intestinal pathogens including *Listeria monocytogenes, Salmonella typhimurium*, and *Campylobacter jejuni* produce flagella for successful host colonization and invasion (82–84). TLR5 signaling in DCs results in activation of the IL-22/23 axis (85). IL-23 then stimulates the secretion of IL-17A by memory T cells and promotes naïve CD4^+^ T cells to differentiate into T_h_17 cells (Figure 1) (86–88). Activation of TLR5 expressed by epithelial cells and DCs plays an important role in clearing pathogenic bacteria such as adherent invasive *E. coli*. Epithelial TLR5 signaling is important in limiting bacterial adherence in the intestine (85).

TLRs can also interact with and effect the signaling of other innate immune receptors to influence T cell function. This is displayed by interactions that occur between TLRs and NOD2. NOD2 is part of the NLR family, is encoded by the Card15 gene, and recognizes Gram-positive and Gram-negative peptidoglycan-derived peptides including muramyl dipeptide (MDP) (89, 90). Activation of NOD2 is associated with altered TLR2 signaling. As stated previously, TLR2 is activated in response to peptidoglycan. Both NOD2 and TLR2 activation result in NF-κB signaling. When NOD2 signaling is stimulated by MDP, TLR2-induced NF-κB signaling and production of IL-12 are inhibited. However, in Card15^−/−^ APCs, NOD2 signaling does not occur but TLR2 signaling can still occur; when treated with peptidoglycan, these mice display higher production of IL-12 by macrophages due to increased NF-κB signaling. This enhances Th1 responses (production of IL-12, IFN-γ, and IL-18) (91). Besides being expressed by APCs, TLR9 and NOD2 are unique because they are both expressed by Paneth cells, a subset of antimicrobial peptide-producing epithelial cells within the crypts of the small intestine. While many antimicrobial peptides are constitutively expressed, signaling by bacterial antigens including flagellin, peptidoglycan, and lipopolysaccharide can further stimulate their production (92).

Enteric pathogens utilize virulence factors to avoid detection by and alter the response of the innate immune system. Two such pathogens are *Salmonella* enterica serovar Typhi and *Salmonella* enterica *serovar Typhimurium*. While *S. Typhi* and *S. Typhimurium* are closely related enteric pathogens, they promote different disease states in humans. *S. Typhi* causes typhoid fever by crossing the intestine and then spreading to systemic organs; on the other hand, *S. Typhimurium* infection is restricted to the intestine and typically causes enteritis (93). *S. Typhi* infection is specific to humans and does not occur in mice (94). This may be due to the ability of mice, but not humans, to produce TLR11 which is activated by flagellin94. Unlike *S. Typhi, S. Typhimurium* can infect multiple hosts including mice. Therefore, *S. Typhimurium* is used to study typhoid-like dissemination in susceptible strains of mice (94, 95). Adaptive B and T cell responses are required to provide protection in mouse model of Salmonella infection. In infected mice, *S. Typhimurium* invades ileal epithelial cells including microfold (M) cells. M cells are specialized epithelial cells that aid in the transcytosis of luminal antigens and microbes into PPs, a region of secondary lymphoid tissue that contains DCs, T cells, and B cells (96). Upon sensing the bacteria, epithelial goblet cells upregulate mucus production, specifically mucin 2, to restrict contact of S. Typhimurium with the epithelial layer (97). To circumvent this, S. Typhimurium expresses flagella and a type III secretion system (T3SS) that is encoded by Salmonella Pathogenicity Island 1 (SPI-1) (84, 93). Effector proteins are injected through the SPI-1 T3SS into epithelial cells to alter their cytoskeletal shape and tight junction integrity. While injection of effectors into M cells promotes optimal bacterial invasion, SPI-1 mutants of S. Typhimurium can still invade M cells but to a lesser extent (Figure 1) (93). Once inside the PP, S. Typhimurium can infect DCs to be transported in a CCR7-dependent manner to MLNs (98). S. Typhimurium then spreads to systemic tissues including the liver and spleen and replicates within phagocytes such as macrophages (93). To avoid degradation within macrophages, S. Typhimurium expresses a second T3SS encoded by Salmonella Pathogenicity Island 2 (99). DCs infected by S. Typhimurium display inhibited antigen presentation which prevents stimulation of naïve T cells (100). S. Typhimurium can also directly infect T cells to inhibit their proliferation and secretion of cytokines including IL-2 and IFN-γ (100).

Another well-studied enteric pathogen, *L. monocytogenes*, alters APC function to influence T cell function. Similar to *S. Typhimurium, L. monocytogenes* replicates within macrophages. Infected macrophages release TNF-α and IL-12 which promote the secretion of IFN-γ from NK cells (101). Secreted IFN-γ then activates macrophages to produce reactive oxygen and nitrogen intermediate to prevent the escape of *L. monocytogenes* from phagosomes and aid in bacterial degradation (102). To promote its growth, *L. monocytogenes* induces production of type I interferons (IFNs). Escape of *L. monocytogenes* from macrophage phagosomes (which occurs due to the production of listeriolysin O, a pore-forming toxin) results in the upregulation of IFN-α and IFN-β by the infected macrophage (103, 104). Increased production of type I IFNs has been associated with increased apoptosis of T cells and greater production of IL-10 to favor bacterial proliferation (104).

Memory T cells also aid with combating infection by *L. monocytogenes* and comprise a major of component of intestinal T cells including tissue effector memory (TEM), central memory (TCM), and resident memory cells (TRM). While TEM and TCM cells both reside in the blood and spleen, TCM can reside within lymphoid tissues and TEM can reside in non-lymphoid tissue (105). TRM cells predominantly reside within the lamina propria and intraepithelial lymphocyte regions of the intestine and provide immune regulation throughout life (106). Upon oral infection with L. monocytogenes, CD103+ DCs acquire and process bacterial antigens. These DCs express CCR7 and travel from the LP via the lymphatics to the MLN (107, 108). In the MLN, DCs present *L. monocytogenes* antigens to naïve CD8 T cells. Activated T cells proliferate and differentiate into early effector cells that further differentiate into either short-lived effector cells or memory precursor effector cells (108). Memory precursor effector cells can differentiate into TEM or TCM and migrate to the intestine (108). DCs release retinoic acid which binds to the retinoic acid receptor of T cells. Upon binding to RA, T cells exhibit enhanced expression integrin α4β7 and CCR9, both of which direct migration to the LP of the small intestine (105, 109). Once at their site of infection, TEM cells express granzyme B. Expression of granzyme B, however, is downregulated in TCM cells (105).

Enteropathogenic *E. coli* (EPEC) and Enterohaemorrhagic *E. coli* (EHEC) are two clinically significant human pathogens of the intestine that are responsible for deaths caused by severe diarrhea. *Citrobacter rodentium* (*C. rodentium*) is a mouse pathogen that shares several clinical pathological mechanisms of EPEC and EHEC and, therefore, serves as a useful model to understand the innate and adaptive immune responses in the gut following infection as well as pathogenesis of IBD (110, 111). The attaching and effacing (A/E) lesion formed by EPEC, EHEC, and *C. rodentium* distinguishes them from other intestinal pathogens and commensal E. coli. A/E lesions form when the pathogenic bacterium binds to the intestinal epithelium, remodels the brush border, and injects effector proteins into the host cell via a T3SS. These effectors then influence the activity of host actin nucleation factors, N-WASP and Arp2/3, to promote actin polymerization. This results in the formation of an actin pedestal that raises the bacterium above neighboring epithelial cells to further assist its pathogenesis (112). Both innate and adaptive immune responses are required to control *C. rodentium* infection since mice deficient in Rag1 succumb to infection (113). *C. rodentium* infection leads to microbiota dysbiosis and the development of colitis (114, 115). Interestingly, microbiota dysbiosis is a key factor that influences the susceptibility of *C. rodentium* infection and immune responses (115, 116). Myd88-dependent TLR2 and TLR4 signaling is stimulated in epithelial and myeloid cells to recognize bacterial PAMPs. DCs and macrophages secrete proinflammatory cytokines including IL-12, IL-6, IL-23, and TNF-α in response to the activation of PRRs. Th17-, Th22-, and ILC3-derived IL-22 plays an important role in regulating *C. rodentium* infection (117, 118). IL-23 is required for provide protection against *C. rodentium* infection in a IL-22 dependent manner117. IL-22-dependent induction of antimicrobial peptide Reg3γ has been suggested to regulate *C. rodentium* infection. However, a recent study shows that Reg3γ^−/−^ mice are equally susceptible to infection. It is possible that another Reg3 family member of antimicrobial peptide compensated for Reg3γ. It remains unclear how IL-22 regulates *C. rodentium* infection. Since IL-22Ra1 is expressed on absorptive cells (enterocytes), secretory cells (goblet, Paneth), and stem cells of intestine, future work should be directed toward understanding the effects of IL-22 on multiple intestinal cell lineages (119).

Yersinia species are Gram-negative bacterium, among them *Y. pestis, Y. pseudotuberculosis*, and *Y. enterocolitica*, are pathogenic to human. *Y. pseudotuberculosis* and *Y. enterocolitica* cause yersiniosis which leads to gastroenteritis and mesenteric lymphadenitis and may be fatal if disseminated into liver and spleen. Similar to Salmonella and other pathogens, Yersinia use T3SS to inject toxin (Yersinia outer protein, Yops) to cell cytoplasm. These Yops (YopE, YopJ, YopH, YopM, YopO, and YopT) disrupt intracellular signaling in macrophages which results into inhibition of cytokines (IL-1β, IL-18, required for innate immune cells recruitment) secretion and phagocytosis. Several studies show that YopE contain a dominant CD8 T cells epitope which is required to confer protection (120–124). Furthermore, CD8 T cells response are also required to provide protection from subsequent infection. CXCR1+ macrophages and/or DC-dependent antigen presentation and local inflammatory conditions are critical for the development of heterogenous population (CD103+ or CD103–) of CD8 TRM cells in the intestine (125).

## Immunity in Response to Cancer

Colorectal cancer (CRC) consistently accounts for many of the cancer-related deaths in men and women. It is the third most common cancer in men and women and the fourth leading cause of cancer-related deaths. Although there are wide variations in global incidence and mortality rates related to CRC, rapidly transitioning countries (indicated by a medium to high human development index) tend to display increased CRC incidence and mortality (122). Over the past few years, the incidence of CRC has been declining in individuals ≥50 years old but is increasing in young adults (126). The onset of colorectal cancer has been associated with various factors including genetics, intestinal microbiota, and immune activity (127–129). Particularly, the release of cytokines by APCs has been shown to influence the development of cancer cells (87, 130, 131).

CRC cells express tumor-associated antigens (TAAs) and tumor-specific antigens (TSAs). While TSAs are unique to tumors, TAAs can be present on normal cells and tumor cells. Common TAAs and TSAs include carcinoembryonic antigens (CEA), Wilm's tumor gene 1 (WT1), Muc1, Her2, and p53 (132–134). DCs can capture bodies of killed tumor cells, process TAAs, and present these antigens via MHC I or MHC II molecules, respectively, to TCRs of CD4^+^ T cells or CD8^+^ T cells (135). APC activation in response to cancer can also occur via the binding of APC-expressed PRRs to tumor-derived DAMPs. DAMPs may be released by tumor cells in response to anti-tumor therapy or stress pathways. With regard to CRC, three of the major DAMPs that stimulate anti-tumorigenic responses of DCs include High Mobility Group Box 1 (HMGB1), extracellular ATP, and calreticulin (CRT). Although typically restricted to the nucleus and cytoplasm, HMGB1 is secreted by necrotic tumor cells. Released HMGB1 can bind to DC-expressed TLR4 to enhance antigen presentation (136, 137). HMGB1 can also signal via DC-expressed RAGE membrane protein to promote NF-κB signaling and DC maturation (138). ATP released from dying tumor cells can bind to the P2XY receptor of DCs. This binding promotes the recruitment of DCs to the tumor stroma. CRT is typically expressed within the endoplasmic reticulum (ER) of tumorigenic cells. Chemotherapeutic agents induce the release of reactive oxygen species and induce ER stress. This promotes the transport of CRT from the ER to the cell surface where CRT serves as a signal for DC-mediated engulfment, degradation, and antigen presentation to cytotoxic CD8^+^ T cells (139). While DCs are vital for antitumor immune responses, cancer cells utilize various mechanisms to evade immune detection. Cancer cells can downregulate TAAs, modulate antigen processing or presentation pathways, release cytokines that promote T_reg_ function, secrete immunosuppressive factors, and express ligands that block immune checkpoints (140). Additionally, immunosuppressive cells within the tumor microenvironment such as tumor-associated macrophages (TAMs), cancer-associated fibroblasts, and T_reg_ cells have been shown to inhibit antitumor immunity (140).

To promote immunity against tumors, DCs and activated T cells secrete multiple cytokines. Two important anti-tumorigenic cytokines are IL-2 and IL-15. These cytokines can induce similar biological effects, and the receptors for these cytokines share similar structural features including a common γ chain and IL-2/IL-15Rβ chain (141, 142). Binding of IL-2 or IL-15 to their receptor results in the activation of Janus kinases (JAK). JAK1 is activated when IL-2 or IL-15 bind to the β chain of the receptor, but JAK3 is activated when IL-2 or IL-15 bind to the or γ chain of the receptor (141). JAKs bind to their corresponding ligand to promote receptor dimerization. Dimerized JAKs phosphorylate each other, and phosphorylated JAKs phosphorylate a conserved tyrosine residue of signal transducers and activators of transcription (STAT) molecules which then enter the nucleus to regulate transcription (141, 143). While IL-2 is secreted mainly by stimulated CD4+ T cells, it is also produced by effector CD8+ T cells, DCs, and NK cells (141, 144–148). Signaling by IL-2 can influence the functions of CD4+ T cells, CD8+ T cells, and NK cells (141, 149–152). IL-15 is expressed on the surface of a range of cell types including monocytes, macrophages, and DCs and influences the activation of DCs, proliferation of CD8+ T cells, and development of NK cells (142, 153–155). IL-2 and IL-15 may play a role in preventing colorectal cancer and have been studied as potential immunotherapy agents (141, 148, 156). However, treatment with these cytokines may have to be supplemented. For instance, survival of melanoma patients treated with IL-2 and a melanoma vaccine displayed increased survival compared to patients who were only treated with IL-2 (157). Studies have also modified the structure of IL-2 to increase its efficiency as an immunotherapeutic agent (158). IL-2 “superkines” have increased affinity for IL-2Rβ and display increased activation of NK and CD8+ T cells. Mice that were injected with melanoma, colon carcinoma, or lung carcinoma cells and then treated with modified IL-2 displayed decreased tumorigenesis compared to mice treated with unmodified IL-2 (158). The onset of colorectal cancer has commonly been studied by the administration of azoxymethane (AOM) combined with dextran sulfate sodium (DSS) to induce colitis-associated carcinoma in mice. Compared to wild-type mice treated with AOM/DSS, Il15^−/−^ mice treated with AOM/DSS display increased tumorigenesis and decreased survival159. CD11c-Il15 mice display reconstituted production of IL-15 in APCs. Unlike Il15^−/−^ mice which display decreased levels of NK and CD8+ T cells, CD11c-Il15 mice display similar levels of NK and CD8+ T cells as wild-type mice. CD11c-Il15 mice also display reduced AOM/DSS-induced tumorigenesis (159). While IL-2 and IL-15 have been shown to reduce the onset of cancer, other studies have shown that they may have little therapeutic effects or may even help promote tumorigenesis (141, 148). Thus, further studies need to be conducted to elucidate the relationship between these cytokines and the onset of cancer.

**Figure 2:**
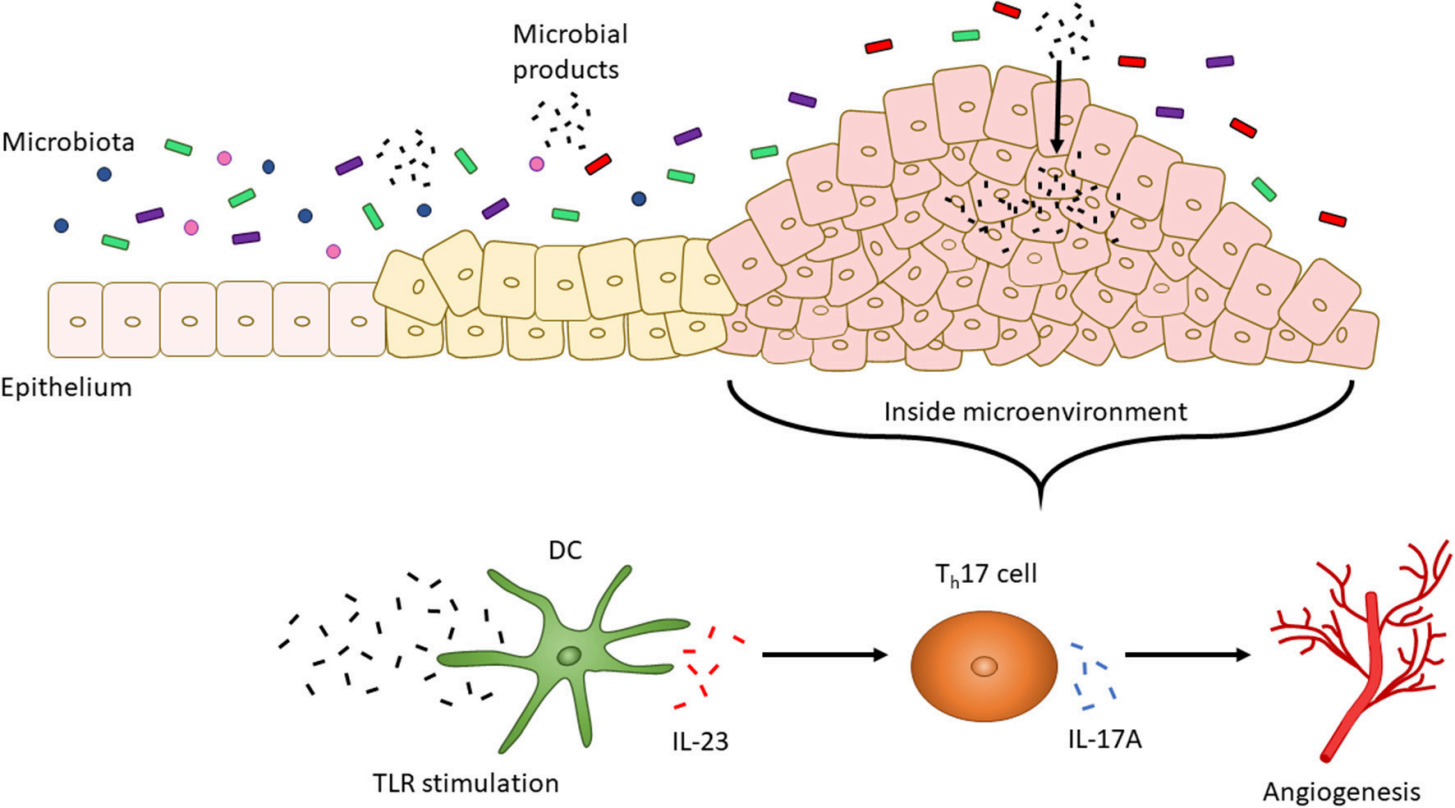
CRC altered microbiota-dependent innate immune signaling facilitates tumorigenesis. The intestinal microbiota changes with the progression of CRC. For instance, increased levels of *F. nucleatum* have been associated with increased severity of CRC. CRC cells also display a “leaky” epithelial layer which allows for the entrance of microbial products into the tumor microenvironment. These products activate TLR signaling by DCs. IL-23 is then secreted by DCs to promote the secretion of IL-17A by T_h_17 cells. IL-17A, in turn, is associated with angiogenesis.

The presence of different T cell classes in the tumor microenvironment has been associated with changes in severity of prognosis. High levels of T_h_17 cells and expression of T_h_17 genes (including *IL17A* and *Rorc*) are associated with a poor prognosis for CRC, but patients with high expression of T_h_1 cytotoxic genes (including *Ccl5, Stat1*, and *Il27*) had improved occurrence of disease-free survival (160, 161). Along with being secreted by T cells, IL-27 is secreted by DCs and macrophages in response to bacterial and parasitic pathogenesis to inhibit T_h_1, T_h_2, and T_h_17 cell development and inflammatory responses (130, 162). Enterocyte-specific knockdown of IL-17RA, a common receptor for IL-17A, IL-17F, and IL-17C resulted in reduced tumor formation, thereby indicating a direct role for IL-17A or other IL-17 family member cytokines in tumorigenesis (163). Upregulation of IL-17A in colon tumors is dependent on upregulated IL-23 signaling by CD11b^+^ cells (164). This increase in IL-23 by DCs, and subsequent increase in IL-17A, may be due to: (1) the microbiota and (2) an impaired epithelial barrier. Compared to the microbiota of healthy individuals, CRC patients possess an altered microbiota distinguishable by the presence of bacteria, such as *Fusobacterium nucleatum*, that promote the proliferation of CRC cells (165). Increased IL-23 may be caused by increased TLR/MyD88 signaling induced by the intestinal microbiota since knockout mice in *Myd88*^−/−^and triple knockout mice in *Tlr2,4,9*^−/−^ display reduced tumor growth. Similarly, mice treated with antibiotics to induce a depleted commensal microbiota displayed reduced tumor size and expression of IL-23 and IL-17A (164). CRC cells have also been shown to be “leaky” since they express defective barrier proteins including mucin 2 and junctional adhesion molecules-A and -B (164). This potentially allows for the increased passage of microbes and microbial products into cancer cells to stimulate TLR signaling and, in turn, IL-23 expression by DCs which then activates T_h_17 cells to release IL-17A (164). Expression of IL-17 has also been linked to tumorigenic processes including the upregulation of proangiogenic factors and neovascularization (Figure 2) (166, 167).

IL-6 is another pro-inflammatory cytokine. IL-6 binding to Janus kinase promotes STAT3 activation (168). STAT3 then enters the nucleus to increase the transcription of anti-apoptotic genes including *Bcl2* and *Mcl1* and metastatic genes including *Mmp1* and *Mmp2* (169–171). Elevated levels of IL-6 have been associated with various cancers including lung, liver, pancreatic, and colorectal (172). Likewise, IL-6 signaling and the subsequent activation of STAT3 have been associated with the translocation of hMSH3, a DNA mismatch repair protein, from the nucleus into the cytosol (173). This translocation allows for the accumulation of tetranucleotide frameshift mutations to promote CRC (173). Some immune cells that produce IL-6 are DCs, macrophages, and T_h_17 cells (87, 131, 174). IL-23 has been shown to induce production of IL-6 by T_h_17 cells (87, 175). In turn, IL-6 aids in the differentiation of T_h_17 cells by promoting expression of IL-21 which induces production of IL-17A via activation of the transcription factors STAT3 and RORγt (176). IL-6 activation also promotes tumorigenesis by altering gene expression for the induction of cell proliferation, progression of the epithelial to mesenchymal transition, and resistance to anti-cancer drugs such as erlotinib (168). Signaling by IL-11, a member of the IL-6 family of cytokines, also promotes the development of CRC by activating STAT3 (177). IL-11 has been identified as a more dominant activator of STAT3 signaling and inducer of CRC than IL-6. Likewise, inhibition of IL-11 signaling has been linked with reduced tumor growth and burden. However, while hematopoietic cells produce IL-11, the secretion of IL-11 by non-hematopoietic cells is more so responsible for the progression of CRC (177). Along with IL-6 and IL-11, elevated levels of IL-22 have been associated with colon cancer. IL-22 has been shown to activate STAT3 signaling and further enhance the development of colon cancer (178, 179). Knockout mice of IL-22 binding protein, a soluble receptor for IL-22, display IL-22-dependent tumor growth (180). ILC3s have been shown to be a major source of IL-22 in mice for promoting tumorigenesis; however, human studies indicate that CCR6^+^ T_h_17 cells are major producers of IL-22 (179, 181). Currently, IL-6 and STAT3 inhibitors are being investigated as potential therapeutic agents to inhibit tumor growth (182).

In addition to STAT3, TNF-α is a critical cytokine for its role in regulating signaling pathways and inflammatory responses, especially in relation to pro- and anti-tumorigenic activity. Like other cytokines, TNF-α is produced by a range of cell types including epithelial cells, macrophages, neutrophils, NK cells, and T cells (183–186). Elevated levels of TNF-α have been associated with increased levels of infiltrating immature myeloid cells which develop into pro-tumorigenic cells such as tumor associated macrophages and neutrophils (TAMs and TANs) once they are exposed to the tumor microenvironment (187, 188). In some cell types, stimulation with TNF-α has been shown to encourage monocyte recruitment to tumors by promoting the expression of monocyte chemoattractant protein-1 (CCL2) (189, 190). Various cell types produce CCL2 including smooth muscle and mesenchymal stromal cells, and other monocytes and macrophages (191–193). TAMs produce cytokines such as IL-10 that inhibit T cell function. IL-10 has been shown to increase the expression of Mgat5, a glycosyltransferase that increases branching of glycoproteins on the surface of CD8^+^ T cells and has been associated with the progression of cancer (194, 195). Increased glycoprotein branching hinders direct T cell interactions with APCs and decreases TCR signaling to promote poor sensitivity to antigens (194). While increased expression of IL-10 produced by TAMs has been correlated with the progression of certain cancers including non-small cell lung carcinoma, the role of IL-10 in promoting CRC is not fully understood as it displays both protective and inflammatory roles (196–198).

Programmed death 1 (PD-1) is expressed on the surface of T cells and interacts with PD-L1, a ligand mainly expressed by macrophages, activated DCs and cancer cells (199). Likewise, cytotoxic T lymphocyte-associated antigen 4 (CTLA4) is expressed on naïve or memory T cells and interacts with CD80/CD86 on the surface of DCs. Although this review will not cover interactions between PD-1 and PD-L1 or CTLA4 and CD80/86 in depth, interactions between PD-1 or CTLA4 and its ligand act to keep T cell activity in check by preventing overactive immune responses (199). Immunotherapy utilizing anti-PD-1 or anti-CTLA4 monoclonal antibodies is effective in fighting the progression of various cancers, and the intestinal microbiota may influence whether certain individuals respond more positively to therapy (199, 200). For instance, increased levels of *Akkermansia muciniphila*, a commensal bacterium, were observed in patients who favorably reacted to treatment (200).

## Conclusion

While much has been done to determine how APCs interact with T cells, several aspects should be expanded. First, how bacteria influence APC interactions with immune cells to combat microbial infections should be further investigated. The interplay between microbiota-derived signaling and the onset of cancers also requires better understanding. Although not widely covered in this review, potential immunotherapies regarding altering APC or microbiota function should be studied. Likewise, a better understanding of how tumors develop strategies to take advantage of immune cells to promote tumorigenesis and dampen T cell responses, especially regarding the PD-1 system, is required. Lastly, more investigation is needed to determine how factors derived from the microbiota, including SCFAs, and intestinal epithelial cells shape APC and immune cell interactions.

## Author Contributions

SG wrote the manuscript and designed the figures. PK edited the text.

### Conflict of Interest Statement

The authors declare that the research was conducted in the absence of any commercial or financial relationships that could be construed as a potential conflict of interest.
